# The Expression of *CARK1* or *RCAR11* Driven by Synthetic Promoters Increases Drought Tolerance in *Arabidopsis thaliana*

**DOI:** 10.3390/ijms19071945

**Published:** 2018-07-03

**Authors:** Hu Ge, Xiaoyi Li, Shisi Chen, Mengru Zhang, Zhibin Liu, Jianmei Wang, Xufeng Li, Yi Yang

**Affiliations:** Key Laboratory of Bio-Resources and Eco-Environment of Ministry of Education, College of Life Sciences, Sichuan University, Chengdu 610065, China; gehu1992@sina.com (H.G.); yiendeavor@gmail.com (X.L.); sschenn@163.com (S.C.); zmrwks@sina.com (M.Z.); lzb2003@163.com (Z.L.); wangjianmei@scu.edu.cn (J.W.); lixufeng0507@gmail.com (X.L.)

**Keywords:** synthetic promoter, transgenic engineering, CARK1, drought, ABA

## Abstract

Drought stress hinders plant growth and development, and abscisic acid (ABA) stimulates plants to respond to drought. Here, to increase plant tolerance to drought, we designed three synthetic promoters (Ap, Dp, ANDp) to determine transcription activity and drought stress resistance in plants resulting from combinations of (1) synthetic promoters and (2) the functional genes *CARK1* (cytosolic ABA receptor kinase 1) and *RCAR11* (regulatory components of ABA receptor 11). Transient expression of *eGFP* and the dual-luciferase assay demonstrated that the basal transcriptional activities of Ap and ANDp were present at low levels under normal conditions, while the synthetic promoters were apparently induced upon either treatment of exogenous ABA or co-transformation with effector DREB2A (dehydration-responsive element binding protein 2A). Analysis of the transgenic plants (Ap:*CARK1*, Dp:*CARK1*, ANDp:*CARK1*, and Dp:*RCAR11*-Ap:*CARK1*) showed that the synthetic promoters Ap, Dp, and ANDp increased the expression of exogenous genes in transgenic plants upon treatment of ABA or d-mannitol. ANDp:*CARK1* and Dp:*RCAR11*-Ap:*CARK1* transgenic plants were sensitive to ABA and d-mannitol during cotyledon greening and root growth. A drought tolerance assay revealed that ANDp:*CARK1* and Dp:*RCAR11*-Ap:*CARK1* exhibited a higher survival rate than others upon drought stress. These results indicate that the combinations ANDp:*CARK1* and Dp:*RCAR11*-Ap:*CARK1* can be used to generate drought stress resistance in plants.

## 1. Introduction

In order to enhance the tolerance of plants to biotic or abiotic stresses, transgenic engineering is used to transfer an exogenous gene to plants, and ultimately cultivate a high-yield crop [1,2,3]. In plants, the 35S cauliflower mosaic virus (CaMV) promoter is used to drive an exogenous gene’s expression [4,5]. Although the 35S CaMV promoter driving gene expression leads to a high expression level in all organs of plants, it often causes the inhibition of the normal growth of plants [6,7]. For example, overexpression of dehydration-responsive element binding protein 1A (*DREB1A*) under the control of the 35S CaMV promoter results in severe growth retardation under normal growing conditions [8]. In *Arabidopsis thaliana*, 35S:FLOWERING LOCUS T-like 1 (*CoFT1*) transgenic plants present an earlier flowering [9]. Overexpression of *TaMyb1D* (*Triticum aestivum* L.), belonging to the R2R3-MYB (myeloblastosis) subfamily, reduces the accumulation of lignin to inhibit normal growth in tobacco plants [6]. Therefore, the selection of proper promoters is important in transgenic engineering. Some natural promoters have been well studied, for example, the *Rab16A* promoter responds to abscisic acid (ABA), salt, and osmotic stresses [10,11,12]. The *COR15A* promoter induces the expression of *COR15A*, a cold-regulated gene [13,14]. To reduce the possibility of inhibiting the overexpression of exogenous genes in plants, scientists use a combination of natural promoters and effector genes to increase plant tolerance to stress [15,16]. However, natural promoters have been found to contain various *cis*-acting elements, which together regulate gene expression under different stresses [17,18,19]. In order to express genes under stress conditions, it is necessary to synthesize the desired promoter which contains specific *cis*-acting elements.

Synthetic promoters are designed by combining *cis*-regulatory sequences with a minimal 35S promoter to regulate ectopic expression and reduce the complexity of the expression pattern of natural promoters [20,21,22]. Synthetic algal promoters (*saps*) have been generated to increase nuclear gene expression in green algae [23]. Most studies have used the *GUS* gene as a reporter gene to detect the function of synthetic promoters [24,25,26]. For example, five tissue-specific synthetic promoters showed different expression efficiencies in various tissues by GUS assays of the transgenic plants [27]. The cold-inducible and tuber-specific activities of chimeric promoters were investigated by quantitative analysis of GUS activity in transgenic potato [28]. Although synthetic promoters have the characteristics of low basic transcription activity and strong inducing ability [27,28,29], it is fewer to use the combination of a synthetic promoter with an effector gene.

In this study, we selected cytosolic ABA receptor kinase 1 (*CARK1*) and regulatory components of ABA receptor 11 (*RCAR11*) as effector genes, which were reported to play a positive role in drought-responsive pathways [30,31,32,33,34]. CARK1 phosphorylates RCAR11 to promote the ABA response, and overexpression of *CARK1* enhances drought tolerance in *Arabidopsis* [33]. *RCAR11*, as one of the ABA receptors, participates in the regulation of the ABA signaling pathway in the nucleus and cytoplasm [30,35]. Based on the function of the effector genes, the design of synthetic promoters was based on elements of *RD29A* (responsive to desiccation 29 A) and *RD29B* (responsive to desiccation 29 B) promoters. The *RD29A* promoter, containing two dehydration-responsive elements (DREs), induces gene expression under drought stress, high salt, and low temperature [17,19,36]. The *RD29B* promoter, with two abscisic acid-responsive elements (ABREs), is involved in the ABA-dependent pathway [29,37,38]. In this study, three stress-inducible promoters were designed and named Ap (containing four tandem repeats of ABRE), Dp (containing two tandem repeats of DRE), and ANDp (containing two tandem repeats of DRE and four tandem repeats of ABRE), respectively. Transient expression of *eGFP* and the dual-luciferase assay demonstrated that these synthetic promoters were functional in *Arabidopsis* protoplasts. The expression levels of *CARK1, RCAR11*, *RD29A*, and *RD29B* were significantly increased in Ap:*CARK1*, Dp:*CARK1*, ANDp:*CARK1*, and Dp:*RCAR11*-Ap:*CARK1* transgenic *Arabidopsis* after ABA or d-mannitol treatment. Cotyledon greening and root length of Ap:*CARK1*, ANDp:*CARK1*, and Dp:*RCAR11*-Ap:*CARK1* transgenic plants were inhibited by exogenous ABA. A drought tolerance assay showed that ANDp:*CARK1* and Dp:*RCAR11*-Ap:*CARK1* transgenic plants exhibited more tolerance to drought stress. Hence, these results indicate that ANDp:*CARK1* and Dp:*RCAR11*-Ap:*CARK1* combinations can be used to generate drought stress resistance in plants.

## 2. Results

### 2.1. Selection of Promoter Fragments

**Figure 1:**
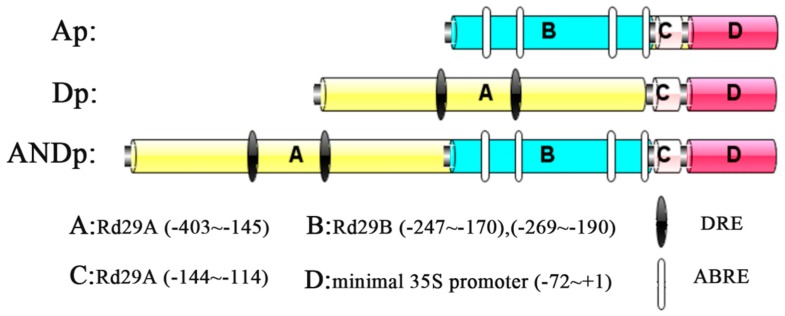
Components of the synthetic promoters.

In this study, the synthetic promoters were designed based on the promoters of *RD29A* and *RD29B*, which contain two DREs and two ABREs, respectively. DRE (A/GCCGAC), as a *cis*-element, regulates osmotic stress-responsive transcription [39,40]. ABRE (ACGTGGC), a major *cis*-acting element, is found in the promoter region of ABA-inducible genes [29,36]. The sequences of *RD29A* and *RD29B* promoters and the *cis*-acting elements were identified from TAIR-Home Page (http://www.arabidopsis.org/index.jsp) and PLANT CARE database (http://bioinformatics.psb.ugent.be/webtools/plantcare/html/), respectively. Based on the functions of *CARK1* and *RCAR11*, the following four sections were selected: A: a fragment of the *RD29A* promoter (−403~−145), including two standard DREs; B: two fragments of the *RD29B* promoter (−247~−170) and (−269~−190), comprising four standard ABREs; C: a fragment of the *RD29A* promoter (−144~−114), which links the regulatory elements and minimal 35S promoter; D: the minimal 35S promoter (−72~+1), including the CAAT frame and the TATA frame. The three designed synthetic promoters are shown in Figure 1.

### 2.2. Confirmation of Transcriptional Activity

**Figure 2:**
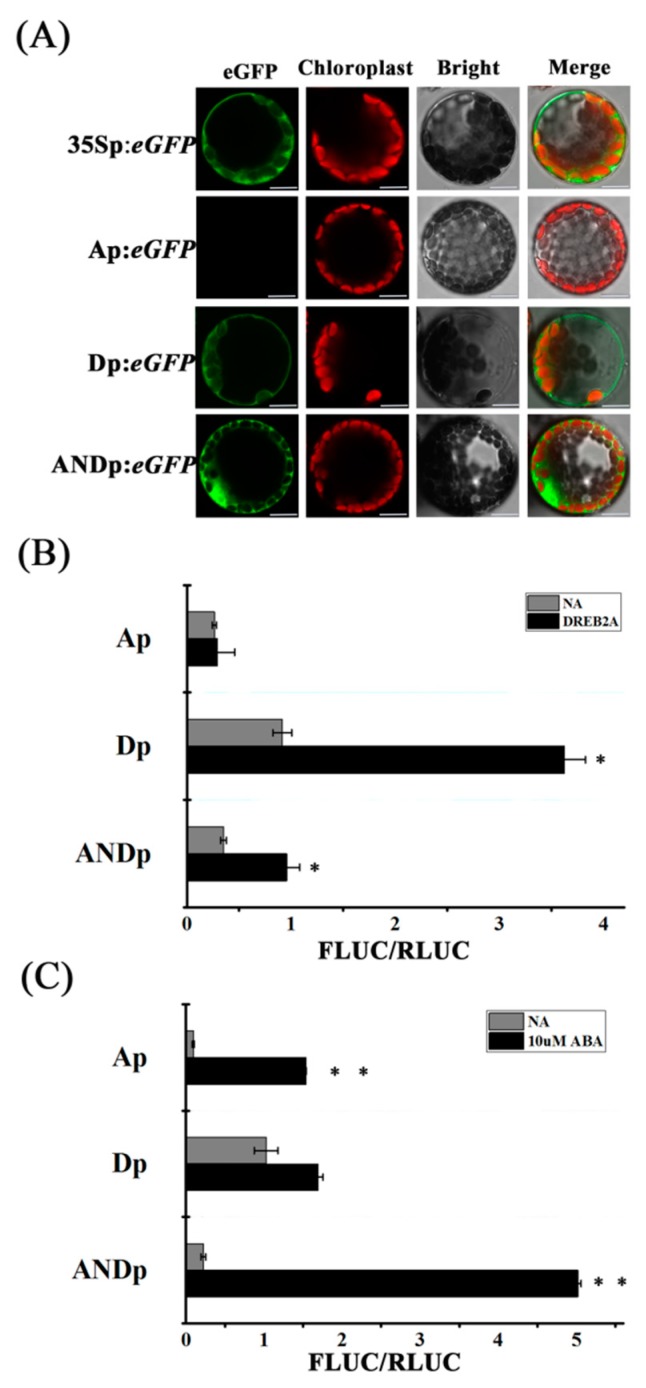
Analysis of transcriptional activities of the synthetic promoters using *Arabidopsis* protoplasts. (**A**) Transactivation of Dp/Ap/ANDp:*eGFP*: the images were taken with a dark field for green fluorescence, a dark field for the red of chloroplast, and a bright field for cell morphology; the images were then merged. Bars = 10 μm; (**B**) Transactivation of the synthetic promoter:*FLUC* by DREB2A (dehydration-responsive element binding protein 2A). Ap:*FLUC*, Dp:*FLUC*, ANDp:*FLUC* were transfected into protoplasts with or without 35S:*DREB2A*; (**C**) Transactivation of the synthetic promoter:*FLUC* by abscisic acid (ABA). The synthetic promoter:*FLUC* plasmid DNA was transfected into protoplasts supplemented with or without 10 μM ABA. Luciferase activity was measured by an LMax II384 luminometer using the Dual-Luciferase Assay Kit after being incubated for 14 h in the dark. RLUC was used as an internal control. Values are mean ± SD (*n* = 3). The experiments were repeated three times (* *p* < 0.05, ** *p* < 0.01, Student’s *t*-test).

To detect transcriptional activities of the synthetic promoters, the transient expression system of *Arabidopsis* protoplasts was used. The signal of green fluorescence from Dp:*eGFP* and ANDp:*eGFP* transfected protoplasts was observed under normal conditions, respectively (Figure 2A). This finding indicates that both Dp and ANDp are able to drive gene expression in plant cells. However, the transfection of Ap failed to induce fluorescence. A possible reason is that the basal transcriptional activity of Ap is low. In addition, these synthetic promoters contained DREs and ABREs. DREB2A (dehydration-responsive element binding protein 2A) regulates DRE-mediated element of genes under osmotic stress conditions in *Arabidopsis* [41,42], and ABRE binding factors (ABFs)/ABRE binding proteins (AREBs) are highly induced by ABA and then interact with ABREs to cause gene expression [43]. To further determine whether the synthetic promoters were induced by DREB2A or ABA, the ratio of FLUC (Firefly luciferase)/RLUC (Renilla luciferase) was analyzed in *Arabidopsis* protoplasts by the dual-luciferase reporter system. Transcription factor *DREB2A* cDNA was cloned into the effector plasmid pBI221, and the synthetized promoter sequences were cloned into the reporter plasmid pGreenII 0800-LUC, respectively. We found that the ratios of FLUC/RLUC in Ap:*FLUC* (0.23) and ANDp:*FLUC* (0.35) were lower than that of Dp:*FLUC* (0.92) under normal conditions (Figure 2B), and the results corroborated those shown in Figure 2C. Ap:*FLUC* was not induced by co-transfection of DREB2A, while Dp:*FLUC* and ANDp:*FLUC* showed 4-fold and 3-fold induction, respectively (Figure 2B). However, in the presence of exogenous ABA, Ap:*FLUC* induced ABA signaling by more than 17-fold, ANDp:*FLUC* by 22-fold, while Dp:*FLUC* had little effect on ABA response (1.5-fold) (Figure 2C). It was noteworthy that the fold increase of FLUC/RLUC of ANDp:*FLUC* was obviously smaller than that of Dp:*FLUC* by co-transfecting with DREB2A, but bigger than Ap:*FLUC* in the presence of exogenous ABA (Figure 2). The reason may be that the distance between the *cis*-acting elements and the TATA box could influence the responsiveness of the promoter, and the DRE-related transcription factors were induced upon exogenous ABA treatment, respectively. These results suggest that these synthetic promoters are functional in *Arabidopsis* protoplasts, for example, Ap activated ABA signaling, and Dp and ANDp responded to DREB2A and ABA. Due to their different transcriptional activities, these synthetic promoters can be used to compare and obtain the optimal combination with *CARK1*. 

### 2.3. Synthetic Promoters Increase the Expression of Drought-Related Genes upon Abscisic Acid (ABA) or d-Mannitol Treatment

**Figure 3:**
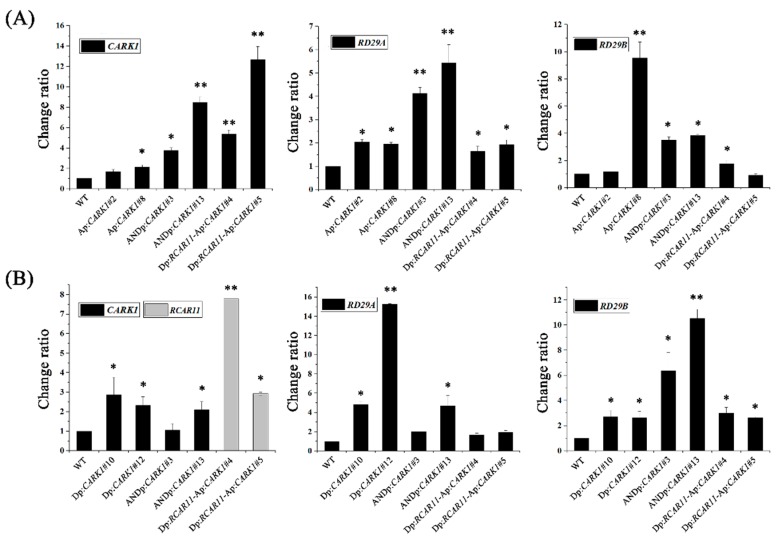
Identification of transgenic lines and analysis of drought-related genes by qRT-PCR. (**A**) ABA. Relative expressional levels of cytosolic ABA receptor kinase 1 (*CARK1*), *RD29A*, and *RD29B* in transgenic plants. Twelve-day-old seedlings were incubated in MS liquid medium with or without 50 μM ABA for 3 h; (**B**) d-Mannitol. Relative expressional levels of *CARK1*, regulatory components of ABA receptor 11 (*RCAR11*), *RD29A*, and *RD29B* in transgenic plants. Twelve-day-old seedlings were incubated in MS liquid medium with or without 200 mM d-mannitol for 2 h. Values are means ± SD (*n* = 3). *ACTIN2/8* was used as an internal control. The change ratio of gene expression in wild type (WT) is 1. The experiments were repeated three times. (* *p* < 0.05, ** *p* < 0.01, ANOVA).

*CARK1* and *RCAR11*, as effector genes, were combined with the synthetic promoters to construct transgenic *Arabidopsis*—Ap:*CARK1*, Dp:*CARK1,* ANDp:*CARK1*, and Dp:*RCAR11*-Ap:*CARK1* (Figure S1). Dp:*RCAR11*-Ap:*CARK1* transgenic *Arabidopsis* was constructed to verify whether this combination of genes and promoters had application value and could increase drought tolerance. The expression of *CARK1* and *RCAR11* was only driven by their upstream synthetic promoters in the Dp:*RCAR11*-Ap:*CARK1* transgenic *Arabidopsis*. Previous results had demonstrated that the synthetic promoters could participate in ABA- or drought-responsive pathways. To determine whether the effector genes were expressed in the transgenic plants, the transcriptional expression level of genes were analyzed by qRT-PCR. There was no significant difference among the expression levels of *CARK1*, *RCAR11* transgenic plants and wild type (WT) under normal conditions, but the transcriptional level of *CARK1* in 35S:*CARK1* transgenic plants was much higher than that of WT (Figure S2). The expression of *CARK1* or *RCAR11* in all transgenic plants was significantly increased after the treatments of ABA and d-mannitol, as shown by qRT-PCR (Figure 3A,B). Furthermore, similar results showed that expression of exogenous *CARK1* or *RCAR11* was induced after treatments of ABA and d-mannitol (Figures S3 and S4). These results indicate that the expression of exogenous *CARK1* or *RCAR11* is at a lower level in these transgenic plants under normal growth conditions but induced by ABA and d-mannitol. To determine the effect of effector genes *CARK1* and *RCAR11* in this study, qRT-PCR was employed to examine the expression levels of ABA- and drought-responsive *RD29A* and *RD29B*. In the presence of exogenous ABA, the expression of *RD29A* and *RD29B* in Ap:*CARK1*, ANDp:*CARK1*, and Dp:*RCAR11*-Ap:*CARK1* transgenic plants were higher than that of WT (Figure 3A). Furthermore, the expression of *RD29A* and *RD29B* in Dp:*CARK1,* ANDp:*CARK1*, and Dp:*RCAR11*-Ap:*CARK1* transgenic plants were also higher than that of WT after d-mannitol treatment (Figure 3B). Taken together, our results demonstrate that Ap, Dp, and ANDp promote exogenous gene expression in transgenic *Arabidopsis* upon treatment of ABA or d-mannitol, and the effector genes *RCAR11* and *CARK1* can be translated normally and then regulate the expression of drought-related genes.

### 2.4. Synthetic Promoters Regulate Plant Growth in Response to ABA

**Figure 4:**
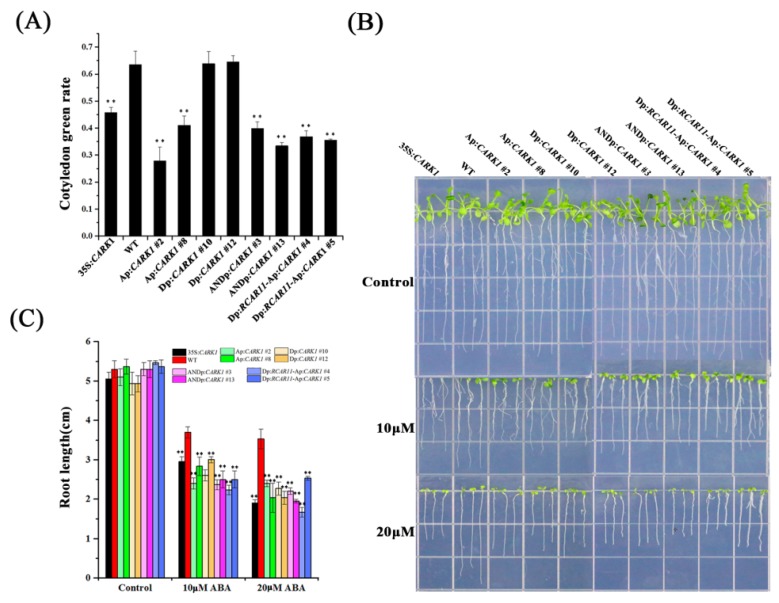
Synthetic promoters regulate plants in response to ABA. (**A**) Cotyledon greening assay. Transgenic plants and WT were in MS medium with or without 0.1 μM ABA, and cotyledon greening rate was recorded after 5 days. The experiments were repeated three times with similar results. Values are means ± SD (*n* > 70) of three independent experiments (** *p* < 0.01, Student’s *t*-test); (**B**) Root length assay. Three-day-old seedlings were transferred to 1/2 MS medium supplemented with or without 10 or 20 μM ABA, and root length measurements were determined after a vertical culture for 7 days; (**C**) Statistical analysis of root length of the different genotypes described in (**B**). Values are means ± SD (*n* = 20). The experiments were repeated three times with similar results (** *p* < 0.01, Student’s *t*-test).

ABA, an important hormone in plants, inhibits seed germination and root growth [44,45,46]. Overexpression of *CARK1* and *RCAR11* driven by the 35S promoter regulates ABA-mediated inhibition of germination, root length, and the expression of ABA-responsive genes [33,35]. To assess whether the expression of *CARK1* or *RCAR11* driven by the synthetic promoters plays a role in plant response to stresses, phenotypic analysis, including cotyledon greening and root growth, was performed. All of the seedlings were similar on the MS (Murashige and Skoog) medium (Figures S5 and S6), while the cotyledon greening rates of all transgenic plants (28~46%) were significantly lower than that of WT (63.5%) in the medium containing 0.1 μM ABA, with the exception of Dp:*CARK1* (64%) (Figure 4A). Other transgenic plants showed similar results upon 0.1 μM ABA treatment (Figure S6). These results reveal that Ap:*CARK1*, ANDp:*CARK1*, and Dp:*RCAR11*-Ap:*CARK1* transgenic plants are sensitive to ABA. To further demonstrate the function of synthetic promoters in ABA signaling, a root growth assay was performed in the presence of exogenous ABA. The root lengths of Ap:*CARK1*, Dp:*CARK1*, ANDp:*CARK1*, and Dp:*RCAR11*-Ap:*CARK1* transgenic plants were no different from 35S:*CARK1* transgenic plants and WT under normal conditions (Figure 4B). However, the root lengths of all the transgenic plants (about 2.2~3.0 cm) were shorter than that of WT (3.7 cm) with 10 μM ABA (Figure 4B,C). In addition, a similar result was found with treatment of 20 μM ABA. Our analysis demonstrates that Dp:*CARK1* is not induced by ABA and leads to plant hyposensitivity to ABA during cotyledon greening, while the expression of Ap:*CARK1*, ANDp:*CARK1*, and Dp:*RCAR11*-Ap:*CARK1* in *Arabidopsis* results in increased sensitivity of plants to ABA during cotyledon greening and root growth.

### 2.5. Synthetic Promoters Enhance Drought Tolerance

**Figure 5:**
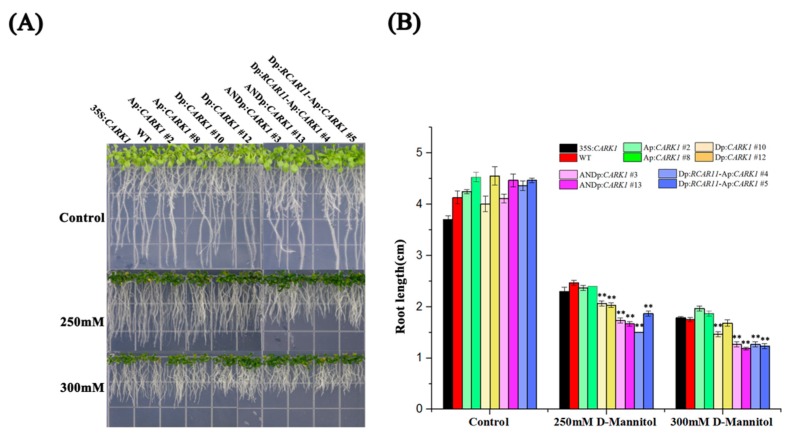

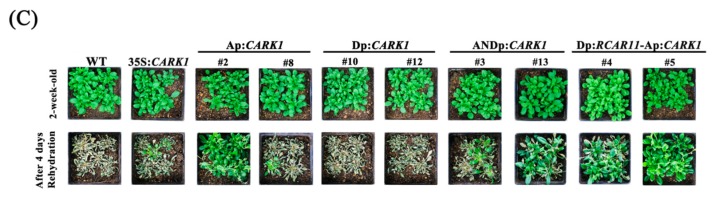
Synthetic promoters involved in the resistance to drought stress. (**A**) Osmotic stress analysis in each transgenic plant with d-mannitol treatment. The seeds were sown on 1/2 MS solid medium supplemented with or without 250, 300 mM d-mannitol; vertical culture and root length measurements were recorded after 14 days; (**B**) Statistical analysis of root length of the different genotypes described in (**A**). Values are means ± SD (*n* = 20). The experiments were repeated three times (** *p* < 0.01, Student’s *t*-test); (**C**) Drought-tolerance assay. Two-week old seedlings were analyzed by withholding water for 12 days and subsequently re-watering for 4 days. The experiments were repeated three times. The photographs show representative measurement.

When plants are subjected to drought stress, dehydration responsive element binding proteins (*DREBs*) interact with DREs to regulate the expression of genes to enhance drought tolerance [47]. d-mannitol was used to mimic osmotic stress to further characterize the role of synthetic promoters. Upon the treatment of 250 or 300 mM d-mannitol, the root lengths of Dp:*CARK1*, ANDp:*CARK1*, and Dp:*RCAR11*-Ap:*CARK1* were significantly shorter than those of 35S:*CARK1*, Ap:*CARK1*, and WT, respectively (Figure 5A,B). This result shows that the synthetic promoters Dp and ANDp increased the *CARK1* expression in transgenic plants upon the treatment of d-mannitol. Further, to compare the drought resistance of transgenic plants more directly, a drought tolerance assay was performed with plants grown in soil. According to the survival rates of all transgenic *Arabidopsis* after withholding water for 12 days and rehydration for 4 days, we found that the survival rates of ANDp:*CARK1* and Dp:*RCAR11*-Ap:*CARK1* transgenic plants were slightly higher than that of Ap:*CARK1* transgenic plants, and were significantly higher than those of 35S:*CARK1* and Dp:*CARK1* transgenic plants (Figure 5C, Table 1). The results of the drought-tolerant assay showed that the survival rates of plants exhibited differences among the different transgenic lines and different experiments (Figure 5C, Table 1). This is arguably because the different insertion sites of the exogenous gene resulted in different transcriptional activities of the synthetic promoters, and the timing of rehydration could have affected the survival rate of plants, respectively. Further experiments were performed, and similar results were obtained (Table S1). Taken together, our results indicate that ANDp:*CARK1* and Dp:*RCAR11*-Ap:*CARK1* transgenic plants exhibited better tolerance to drought stress than other transgenic plants.

## 3. Discussion

Altering the expression of genes affects plant stress tolerance, including salinity and drought tolerance [4,12]. In the study, we selected sequences in the regulatory regions of *RD29A* and *RD29B* genes based on the functions of *CARK1* and *RCAR11* and constructed three synthetic promoters (Ap, Dp, ANDp). The expression of *FLUC* in the protoplasts transformed with Ap:*FLUC* and the transcript abundance of *CARK1* in Ap:*CARK1* transgenic plants were low, but they significantly increased after treatment of ABA (Figure 2 and Figure 3, Figures S2–S4, S7 and S8). On the other hand, the expression of *FLUC* in the protoplasts co-transformed with Ap:*FLUC* was not changed in the presence of the effector DREB2A (Figure 2B), and the root growth of Ap:*CARK1* transgenic plants was insensitive to osmotic stress (d-mannitol) (Figure 5A,B). These findings show that the synthetic promoter Ap had a low basal transcriptional activity and only responded to the ABA. The expression of *FLUC* in the protoplasts transformed with Dp:*FLUC* was induced after co-transformation with DREB2A, and the transcript abundance of *CARK1* in Dp:*CARK1* transgenic plants was increased after the treatment of d-mannitol (Figure 2 and Figure 3 and Figure S3). These results reveal that the synthetic promoter Dp responded to drought stress signals. However, the expression of *FLUC* in the protoplasts transformed with ANDp:*FLUC* were induced upon the treatment of ABA and co-transformation with DREB2A, and the expression of *CARK1* in ANDp:*CARK1* transgenic plants was induced upon the treatment of ABA or d-mannitol (Figure 2 and Figure 3, Figures S3 and S4), showing that the synthetic promoter ANDp was involved in both ABA and drought signaling. Taken together, our results indicate that the synthetic promoters (Ap, Dp, ANDp) were functional in plant cells.

The synthetic promoter Dp does not contain ABREs, but the expression of *FLUC* in the protoplasts transformed with Dp:*FLUC* was slightly increased, and the root growth of Dp:*CARK1* transgenic plants was significantly inhibited in the presence of exogenous ABA (Figure 2C and Figure 4B,C), which is because DREs function as coupling elements of ABRE in response to ABA [29]. It is worth noting that ANDp:*CARK1* transgenic plants were more drought tolerant than Ap:*CARK1*, Dp:*CARK1*, and 35S:*CARK1* transgenic plants (Figure 5C, Table 1 and Table S1), indicating that the expression of *CARK1* under control of ANDp was more effective than Ap, Dp, and 35S promoters. Hence, we could conclude that the appropriate expression of the gene was sufficient to cope with stress. Meanwhile, Dp:*RCAR11*-Ap:*CARK1* and ANDp:*CARK1* transgenic plants had similar drought resistance phenotypes (Figure 4 and Figure 5, Table 1 and Table S1), which provided a more advantageous way to increase tolerance to different stresses and reduce energy consumption in the stage of development and growth in plants.

The comparison of three synthetic promoters (Ap, Dp, ANDp) and five transgenic *Arabidopsis* lines (Ap:*CARK1*, Dp:*CARK1*, ANDp:*CARK1*, 35S:*CARK1*, and Dp:*RCAR11*-Ap:*CARK1*) indicated that the transcriptional activity of ANDp was significantly higher than that of Ap and Dp upon the treatment of ABA or co-transfection with DREB2A (Figure 2). Although all three synthetic promoters responded to specific stress, the synthetic promoters Ap and ANDp minimally initiated gene expression under normal conditions (Figure 3). The expression of *CARK1* under the control of Ap or ANDp in plants was more sensitive to ABA (Figure 4 and Figure S6). The growth of all the transgenic plants had no significant difference under normal conditions (Figures S9 and S10, Table S4), but the survival rate under drought stress demonstrated that ANDp:*CARK1* and Dp:*RCAR11*-Ap:*CARK1* transgenic plants exhibited stronger tolerance to drought than other transgenic plants (Table 1 and Table S1). In summary, our results revealed that the combinations ANDp:*CARK1* and Dp:*RCAR11*-Ap:*CARK1* transgenic plants not only had the least impact on plants under normal growth conditions but also imparted strong tolerance to drought stress in the plants. Hence, the combinations ANDp:*CARK1* and Dp:*RCAR11*-Ap:*CARK1* can be used to generate drought stress resistance in plants*.*

## 4. Materials and Methods

### 4.1. Plant Materials and Growth Conditions

The *Arabidopsis thaliana* wild type (WT) and transgenic plants used in this study are Columbia (Col-0) ecotype. To construct the *Arabidopsis* transgenic plants Ap:*CARK1*, Dp:*CARK1*, ANDp:*CARK1*, and Dp:*RCAR11*-Ap:*CARK1* (Figure S1), the following steps were taken. The 35S:*CARK1* was obtained from our laboratory as positive control. The 35S promoter was replaced by the synthetic promoters (Ap, Dp, ANDp) and inserted into the pBI121-35S:*CARK1* vector with *Hind*III and *Xba*I sites. The DNA sequence of Dp:*RCAR11*-nos was obtained by PCR and inserted into the pBI121-Ap:*CARK1* vector at the *Hind*III site by the recombination method. The constructs were introduced into the GV3101 strain of *Agrobacterium tumefaciens*. The floral dip method was used in this study [48]. The T1 generation of transgenic plants that grew well (with green leaves and long primary roots) on kanamycin/MS solid medium were transferred to soil to obtain T2 seeds. The T2 generation was also screened on MS supplemented with kanamycin, and the single-copy genes displayed a Mendelian segregation ratio (3:1). The mRNA levels were identified with qRT-PCR assays. T3 homozygous seeds of the transgenic plants were used for further analysis. The primers that were used in this assay are listed in Table S2.

For nonsterile culture, seeds were grown in soil (vermiculite:nutrient soil, 1:2) in greenhouses at 22 °C under 60% humidity with 16 h light (250 μmol m^−2^ s^−1^)/8 h dark cycles. For sterile culture, surface-sterilized seeds were sown on MS medium containing 2% (*w*/*v*) sucrose and 0.8% (*w*/*v*) agar, pH 5.7 [49].

### 4.2. Protoplast Isolation and Transient Activation Assay

Preparation of *Arabidopsis* protoplasts of the wild-type plants and subsequent transfection of protoplasts were performed as described [50]. For analysis of transient expression of *eGFP*, the synthetic promoters Ap, Dp, and ANDp were generated by replacing the 35S promoter in the vector pBI221-*eGFP* via *Hind*III and *Xba*I sites. The vectors 35S:*eGFP*, Dp:*eGFP*, Ap:*eGFP*, and ANDp:*eGFP* were used at 2 μg per transfection. After incubation about 14 h, cells with *eGFP* fluorescence were observed and imaged with the confocal laser-scanning microscope (Leica TCS SP5 II systemT, Leica, Germany).

For the dual-luciferase reporter system assay [51], Ap, Dp, and ANDp were inserted into the vector pGreenII 0800-LUC at *Hind*III and *BamH*I sites. The internal control construct 35S:*RLUC* and activator construct 35S:*DREB2A* were generated as described [40]. The vector 35S:*DREB2A* was used at 2 μg per transfection, and the vectors Dp:*FLUC*, Ap:*FLUC*, and ANDp:*FLUC* were used at 2 μg per transfection. When indicated, 10 μM ABA was added into the incubation buffer immediately after transfection. After incubation for about 14 h, luciferase activity was measured by an LMax II384 luminometer (Molecular Devices, Bad Wildbad, Germany) using the Dual-Luciferase Assay Kit (Vigorous, Beijing, China). Relative FLUC activity was calculated by normalizing against the RLUC activity, and the data presented are the averages of three biological replicates. Primers used in this study are listed in Table S3.

### 4.3. Analysis of Quantitative Real Time-PCR (qRT-PCR)

Total RNA was extracted from 12-day-old *Arabidopsis* seedlings treated with 50 μM ABA for 3 h or 200 mM d-mannitol for 2 h using TRIzol RNA reagent (TaKaRa, Dalian, China). The cDNA was synthesized using PrimeScript RT reagent Kit with gDNA Eraser (TaKaRa, China) following the manufacturer’s instruction. qRT-PCR analysis was carried out on a CFX96 TouchTM Real-Time PCR detection system (Bio-Rad, Hercules, California, CA, USA) with iQTM SYBR Green Supermix and gene-specific primers. All reactions were performed in triplicate with the following cycling conditions: 95 °C for 3 min; 40 cycles each at 95 °C for 10 s and 56 °C for 30 s, and 72 °C for 20 s. Actin 2/8 was used as an internal control. All primers are shown in Table S2.

### 4.4. Phenotype Analysis

For the cotyledon greening assay, about 70 seeds from the wild type and the transgenic plants were sterilized and sown on MS medium supplemented with the indicated 0.1 μM ABA after stratification. Cotyledon greening was recorded as the percentage of seeds with green expanded cotyledons and the first pair of true leaves at 5 days.

For the root growth assay, about 100 seeds from each line were first germinated vertically on MS medium for 3 days after stratification. Then, five seedlings of each line sharing similar root length were transferred to 1/2 MS medium supplemented with 0, 10, 20 μM ABA in the vertical position. The root length was determined after transfer for 7 days. For the drought tolerance assay, six seeds from each line were germinated vertically on 1/2 MS medium with 0, 200, 250 mM d-mannitol, and the root length was determined after transfer for about 14 days.

For the drought-tolerance test, 2-week-old seedlings were subjected to drought stress treatment by withholding water for 12 days. Then, 4 days after rehydration, the morphological changes of plants were recorded.

### 4.5. Statistical Analysis

Data are represented as means ± SD. Statistical analysis was performed using Student’s *t*-test. Values of *p* < 0.05 were considered significant, and values of *p* < 0.01 were considered more significant.

## Figures and Tables

**Table 1:** Survival rate of drought-tolerance assay. Three independent measurements are shown.

	WT	35S:*CARK1*	Ap:*CARK1*		Dp:*CARK1*		ANDp:*CARK1*		Dp:*RCAR11*-Ap:*CARK1*	
			#2	#8	#10	#12	#3	#13	#4	#5
1	0	6%	2%	0	0	0	80%	95%	100%	90%
2	4%	0	11%	7.5%	0	80%	9%	90%	100%	55.5%
3	0	16.7%	100%	10%	0	0	20%	85%	90%	100%

## Supplementary Materials

Supplementary materials can be found at http://www.mdpi.com/1422-0067/19/7/1945/s1.

## References

[1] Fujita Y., Fujita M., Satoh R., Maruyama K., Parvez M.M., Seki M., Hiratsu K., Ohme-Takagi M., Shinozaki K., Yamaguchi-Shinozaki K. (2005). AREB1 is a transcription activator of novel ABRE-dependent ABA signaling that enhances drought stress tolerance in *Arabidopsis*. Plant Cell.

[2] Kurek I., Chang T.K., Bertain S.M., Madrigal A., Liu L., Lassner M.W., Zhu G. (2007). Enhanced Thermostability of Arabidopsis Rubisco activase improves photosynthesis and growth rates under moderate heat stress. Plant Cell.

[3] Zhou Y., Tao Y., Zhu J., Miao J., Liu J., Liu Y., Yi C., Yang Z., Gong Z., Liang G. (2017). *GNS4*, a novel allele of *DWARF11*, regulates grain number and grain size in a high-yield rice variety. Rice.

[4] Yang L., Wu L., Chang W., Li Z., Miao M., Li Y., Yang J., Liu Z., Tan J. (2017). Overexpression of the maize E3 ubiquitin ligase gene *ZmAIRP4* enhances drought stress tolerance in *Arabidopsis*. Plant Physiol. Biochem..

[5] Dong X., Zhao Y., Ran X., Guo L., Zhao D.G. (2017). Overexpression of a New Chitinase Gene *EuCHIT2* EnhancesResistance to *Erysiphe cichoracearum* dc in Tobacco Plants. Int. J. Mol. Sci..

[6] Wei Q., Zhang F., Sun F., Luo Q., Wang R., Hu R., Chen M., Chang J., Yang G., He G. (2017). A wheat MYB transcriptional repressor TaMyb1D regulates phenylpropanoid metabolism and enhances tolerance to drought and oxidative stresses in transgenic tobacco plants. Plant Sci..

[7] Zhai N., Jia H., Liu D., Liu S., Ma M., Guo X., Li H. (2017). *GhMAP3K65*, a Cotton Raf-Like MAP3K Gene, Enhances Susceptibility to Pathogen Infection and Heat Stress by Negatively Modulating Growth and Development in Transgenic *Nicotiana benthamiana*. Int. J. Mol. Sci..

[8] Kasuga M., Liu Q., Miura S., Shinozaki K., Yamaguchi-Shinozaki K. (1999). Improving Plant Drought, Salt and Freezing Tolerance by Gene Transfer of a Single Stress-Inducible Transcription Factor. Nat. Biotechnol..

[9] Lei H., Su S., Ma L., Wen Y., Wang X. (2017). Molecular cloning and functional characterization of *CoFT1*, a homolog of *FLOWERING LOCUS T (FT)* from *Camellia oleifera*. Gene.

[10] Mundy J., Yamaguchi-Shinozaki K., Chua N.H. (1990). Nuclear proteins bind conserved elements in the abscisic acid-responsive promoter of a rice *rab* gene. Proc. Natl. Acad. Sci. USA.

[11] Ono A., Izawa T., Chua N.H., Shimamoto K. (1996). The *rab16B* Promoter of Rice Contains Two Distinct Abscisic Acid-Responsive Elements. Plant Physiol..

[12] RoyChoudhury A., Roy C., Sengupta D.N. (2007). Transgenic tobacco plants overexpressing the heterologous *lea* gene *Rab16A* from rice during high salt and water deficit display enhanced tolerance to salinity stress. Plant Cell Rep..

[13] Baker S.S., Wilhelm K.S., Thomashow M.F. (1994). The 5’-region of *Arabidopsis thaliana cor15a* has cis-acting elements that confer cold-, drought- and ABA-regulated gene expression. Plant Mol. Biol. Rep..

[14] Li M., Wang X., Cao Y., Liu X., Lin Y., Ou Y., Zhang H., Liu J. (2013). Strength comparison between cold-inducible promoters of *Arabidopsis cor15a* and *cor15b* genes in potato and tobacco. Plant Physiol. Biochem..

[15] Behnam B., Kikuchi A., Celebi-Toprak F., Kasuga M., Yamaguchi-Shinozaki K., Watanabe K.N. (2007). *Arabidopsis* rd29A:*DREB1A* enhances freezing tolerance in transgenic potato. Plant Cell Rep..

[16] Zhao Y., Chan Z., Gao J., Xing L., Cao M., Yu C., Hu Y., You J., Shi H., Zhu Y. (2016). ABA receptor PYL9 promotes drought resistance and leaf senescence. Proc. Natl. Acad. Sci. USA.

[17] Yamaguchi-Shinozaki K., Shinozaki K. (1994). A novel cis-acting element in an *Arabidopsis* gene is involved in responsiveness to drought, low-temperature, or high-salt stress. Plant Cell.

[18] Rerksiri W., Zhang X., Xiong H., Chen X. (2013). Expression and promoter analysis of six heat stress-inducible genes in rice. Sci. World J..

[19] Msanne J., Lin J., Stone J.M., Awada T. (2011). Characterization of abiotic stress-responsive *Arabidopsis thaliana RD29A* and *RD29B* genes and evaluation of transgenes. Planta.

[20] Venter M. (2007). Synthetic promoters: Genetic control through cis engineering. Trends Plant Sci..

[21] Shokouhifar F., Zamani M.R., Motallebi M., Mousavi A., Malboobi M.A. (2011). Construction and functional analysis of pathogen-inducible synthetic promoters in *Brassica napus*. Biol. Plant..

[22] Liu W., Stewart C.N. (2016). Plant synthetic promoters and transcription factors. Curr. Opin. Biotechnol..

[23] Scranton M.A., Ostrand J.T., Georgianna D.R., Lofgren S.M., Li D., Ellis R.C., Carruthers D.N., Dräger A., Masica D.L., Mayfield S.P. (2016). Synthetic promoters capable of driving robust nuclear gene expression in the green alga *Chlamydomonas reinhardtii*. Algal Res..

[24] Ganguly M., Roychoudhury A., Sarkar S.N., Sengupta D.N., Datta S.K., Datta K. (2011). Inducibility of three salinity/abscisic acid-regulated promoters in transgenic rice with *gusA* reporter gene. Plant Cell Rep..

[25] Du L., Lou Q., Zhang X., Jiao S., Liu Y., Wang Y. (2013). Construction of Flower-specific Chimeric Promoters and Analysis of Their Activities in Transgenic *Torenia*. Plant Mol. Biol. Rep..

[26] Zhu Z., Gao J., Yang J.X., Wang X.Y., Ren G.D., Ding Y.L., Kuai B.K. (2015). Synthetic promoters consisting of defined cis-acting elements link multiple signaling pathways to probenazole-inducible system. J. Zhejiang Univ. Sci. B.

[27] Wang R., Zhu M., Ye R., Liu Z., Zhou F., Chen H., Lin Y. (2015). Novel green tissue-specific synthetic promoters and cis-regulatory elements in rice. Sci. Rep..

[28] Zhu Q., Song B., Zhang C., Ou Y., Xie C., Liu J. (2015). Construction and functional characteristics of tuber-specific and cold-inducible chimeric promoters in potato. Plant Cell Rep..

[29] Nakashima K., Fujita Y., Katsura K., Maruyama K., Narusaka Y., Seki M., Shinozaki K., Yamaguchi-Shinozaki K. (2006). Transcriptional regulation of ABI3- and ABA-responsive genes including *RD29B* and *RD29A* in seeds, germinating embryos, and seedlings of *Arabidopsis*. Plant Mol. Biol..

[30] Park S.Y., Fung P., Nishimura N., Jensen D.R., Fujii H., Zhao Y., Lumba S., Santiago J., Rodrigues A., Chow T.F. (2009). Abscisic Acid Inhibits Type 2C Protein Phosphatases via the PYR/PYL Family of START Proteins. Science.

[31] Nishimura N., Sarkeshik A., Nito K., Park S.Y., Wang A., Carvalho P.C., Lee S., Caddell D.F., Cutler S.R., Chory J. (2010). PYR/PYL/RCAR family members are major in-vivo ABI1 protein phosphatase 2C-interacting proteins in Arabidopsis. Plant J..

[32] Dorosh L., Kharenko O.A., Rajagopalan N., Loewen M.C. (2013). Molecular Mechanisms in the Activation ofAbscisic Acid Receptor PYR1. PLoS Comput. Biol..

[33] Zhang L., Li X.Y., Li D.K., Sun Y.N., Li Y., Luo Q., Liu Z.B., Wang J.M., Li X.F., Zhang H. (2018). *CARK1* mediates ABA signaling by phosphorylation of ABA receptors. Cell Discov..

[34] Gonzalez-Guzman M., Pizzio G.A., Antoni R., Vera-Sirera F., Merilo E., Bassel G.W., Fernandez M.A., Holdsworth M.J., Perez-Amador M.A., Kollist H. (2012). *Arabidopsis* PYR/PYL/RCAR Receptors Play a Major Role in Quantitative Regulation of Stomatal Aperture and Transcriptional Response to Abscisic Acid. Plant Cell.

[35] Park E., Kim T.H. (2017). Production of ABA responses requires both the nuclear and cytoplasmic functional involvement of PYR1. Biochem. Biophys. Res. Commun..

[36] Bihmidine S., Lin J., Stone J.M., Awada T., Specht J.E., Clemente T.E. (2013). Activity of the *Arabidopsis* RD29A and RD29B promoter elements in soybean under water stress. Planta.

[37] Koornneef M., Jorna M.L., Brinkhorst-van der Swan D.L.C., Karssen C.M. (1982). The isolation of abscisic acid (ABA) deficient mutants by selection of induced revertants in non-germinating gibberellin sensitive lines of *Arabidopsis thaliana* (L.) heynh. Theoretical Appl. Genet..

[38] Virlouvet L., Ding Y., Fujii H., Avramova Z., Fromm M. (2014). ABA signaling is necessary but not sufficient for *RD29B* transcriptional memory during successive dehydration stresses in *Arabidopsis thaliana*. Plant J..

[39] Yamaguchi-Shinozaki K., Shinozaki K. (2005). Organization of cis-acting regulatory elements in osmotic- and cold-stress-responsive promoters. Trends Plant Sci..

[40] Nakashima K., Ito Y., Yamaguchi-Shinozaki K. (2009). Transcriptional regulatory networks in response to abiotic stresses in Arabidopsis and grasses. Plant Physiol..

[41] Sakuma Y., Maruyama K., Osakabe Y., Qin F., Seki M., Shinozaki K., Yamaguchi-Shinozaki K. (2006). Functional analysis of an *Arabidopsis* transcription factor, DREB2A, involved in drought-responsive gene expression. Plant Cell.

[42] Kim J.S., Mizoi J., Yoshida T., Fujita Y., Nakajima J., Ohori T., Todaka D., Nakashima K., Hirayama T., Shinozaki K. (2011). An ABRE promoter sequence is involved in osmotic stress-responsive expression of the *DREB2A* gene, which encodes a transcription factor regulating drought-inducible genes in Arabidopsis. Plant Cell Physiol..

[43] Zhao B.Y., Hu Y.F., Li J.J., Yao X., Liu K.D. (2016). BnaABF2, a bZIP transcription factor from rapeseed (*Brassica napus* L.), enhances drought and salt tolerance in transgenic *Arabidopsis*. Bot Stud..

[44] Holdsworth M.J., Finch-Savage W.E., Grappin P., Job D. (2008). Post-genomics dissection of seed dormancy and germination. Trends Plant Sci..

[45] Zhang L., Hu Y., Yan S., Li H., He S., Huang M., Li L. (2012). ABA-mediated inhibition of seed germination is associated with ribosomal DNA chromatin condensation, decreased transcription, and ribosomal RNA gene hypoacetylation. Plant Mol. Biol..

[46] Luo X., Chen Z., Gao J., Gong Z. (2014). Abscisic acid inhibits root growth in Arabidopsis through ethylene biosynthesis. Plant J..

[47] Agarwal P.K., Agarwal P., Reddy M.K., Sopory S.K. (2006). Role of DREB transcription factors in abiotic and biotic stress tolerance in plants. Plant Cell Rep..

[48] Clough S.J., Bent A.F. (1998). Floral dip: A simplified method for *Agrobacterium*-mediated transformation of *Arabidopsis thaliana*. Plant J..

[49] Murashige T., Skoog F. (1962). A Revised Medium for Rapid Growth and Bio Assays with Tobacco Tissue Cultures. Physiologia Plantarum.

[50] Yoo S.D., Cho Y.H., Sheen J. (2007). Arabidopsis mesophyll protoplasts: A versatile cell system for transient gene expression analysis. Nat. Protoc..

[51] Hellens R.P., Allan A.C., Friel E.N., Bolitho K., Grafton K., Templeton M.D., Karunairetnam S., Gleave A.P., Laing W.A. (2005). Transient expression vectors for functional genomics, quantification of promoter activity and RNA silencing in plants. Plant. Methods.

