# Supplementary material for: The Expression of CARK1 or RCAR11 Driven by Synthetic Promoters Increases Drought Tolerance in Arabidopsis thaliana
Source: Int J Mol Sci. 2018 Jul 3;19(7):1945. doi: 10.3390/ijms19071945 (PMC6073707; doi:10.3390/ijms19071945)
Supplement: Supplementary file 1 [file ijms-19-01945-s001.pdf]

International Journal of
Molecular Sciences

MDPI

Supplementary

The Expression of *CARK1* or *RCAR11* Driven by Synthetic Promoters Increases Drought Tolerance in *Arabidopsis thaliana*

Hu Ge, Xiaoyi Li, Shisi Chen, Mengru Zhang, Zhibin Liu, Jianmei Wang, Xufeng Li and Yi Yang *

Key Laboratory of Bio-Resources and Eco-Environment of Ministry of Education, College of Life Sciences, Sichuan University, Chengdu 610065, China; gehu1992@sina.com (H.G.); yiendeavor@gmail.com (X.L.); sschenn@163.com (S.C.); zmrwks@sina.com (M.Z.); lzb2003@163.com (Z.L.); wangjianmei@scu.edu.cn (J.W.); lixufeng0507@gmail.com (X.L.)

* Correspondence: yangyi528@scu.edu.cn

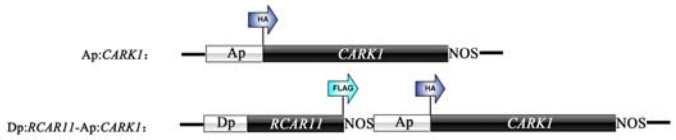

Figure S1. Construction of vectors for transgenic plants. Construction of vectors of Dp:*CARK1* and ANDp:*CARK1* transgenic plants were the same as Ap:*CARK1* transgenic plants.

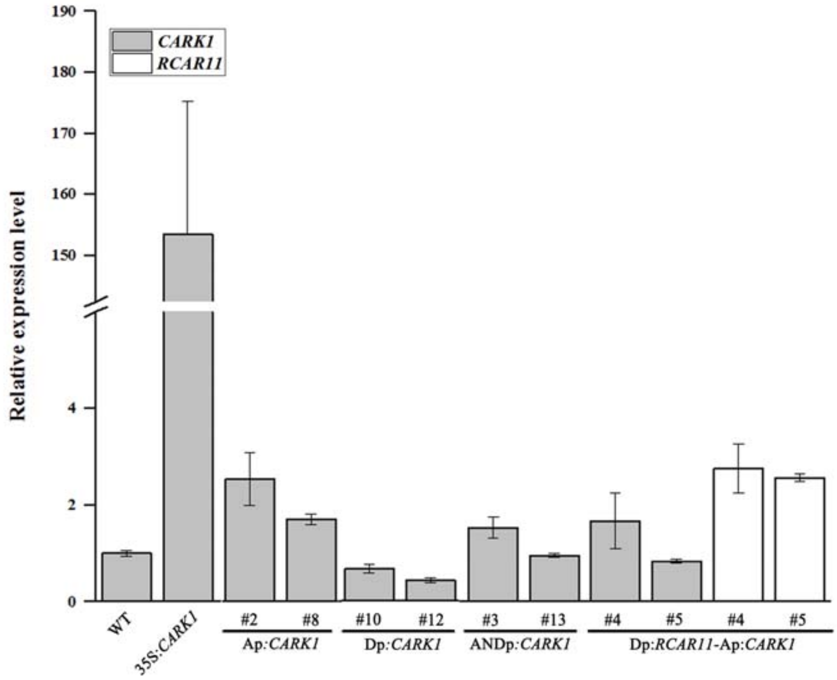

Figure S2. Relative expression levels of effector genes of transgenic plants under normal conditions were determined by qRT-PCR analysis.

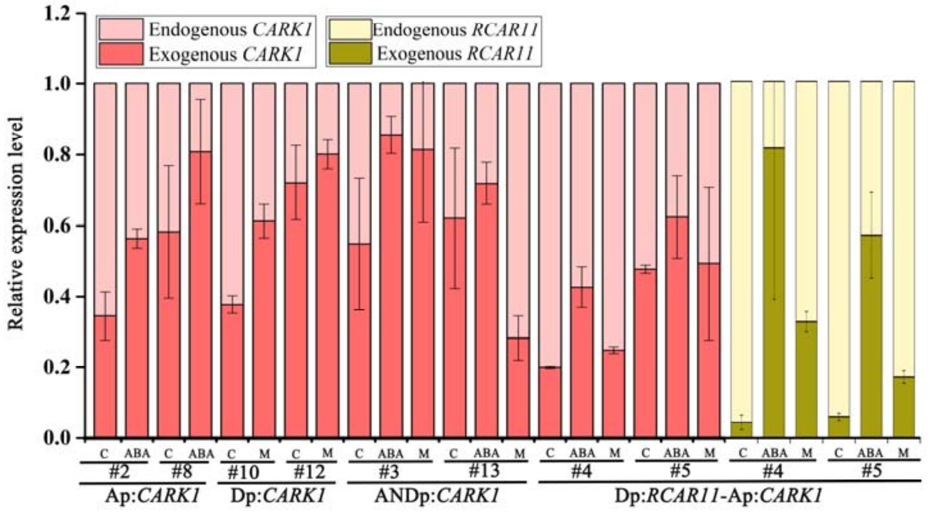

Figure S3. Expression levels of *CARK1* and *RCAR11* under the treatment of ABA or D-mannitol by qRT-PCR analysis. Twelve-day-old seedlings were incubated in MS liquid medium with or without 50 μM ABA for 3 h and 200 mM D-mannitol for 2 h. The total *CARK1* and *RCAR11* were amplified by primers F and R, respectively. The exogenous *CARK1* was amplified by primers F(HA) and R. The exogenous *RCAR11* was amplified by primers F and R(FLAG). *ACTIN2/8* was used as an internal control. The expression level of total *CARK1* or *RCAR11* was 1.

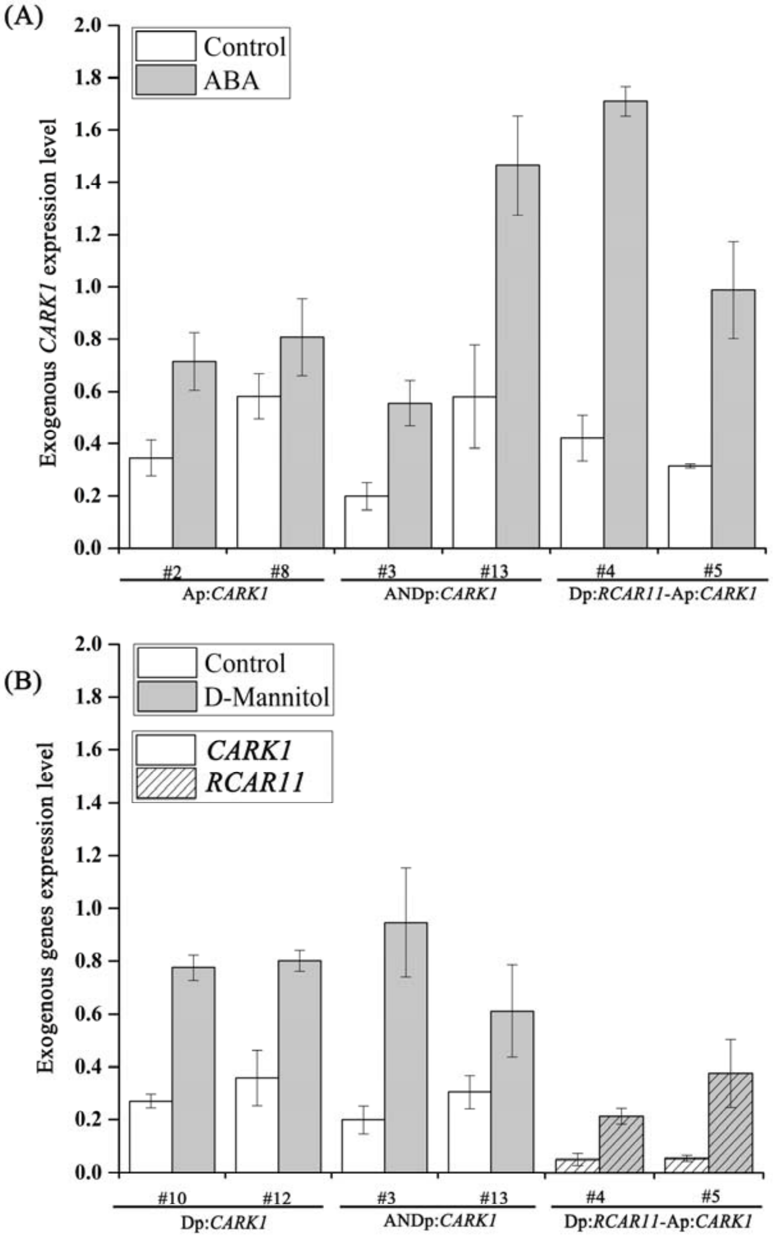

Figure S4. Expression levels of exogenous *CARK1* and *RCAR11* under the treatment of ABA or D-mannitol by qRT-PCR analysis. **(A) ABA** 12-day-old seedlings were incubated in MS liquid medium with or without 50 μM ABA for 3 h. **(B) D-Mannitol** 12-day-old seedlings were incubated in MS liquid medium with or without 200 mM D-mannitol for 2 h. The exogenous *CARK1* was amplified by primers F(HA) and R(CARK1), and the exogenous *RCAR11* was amplified by primers F(RCAR11) and R(FLAG).

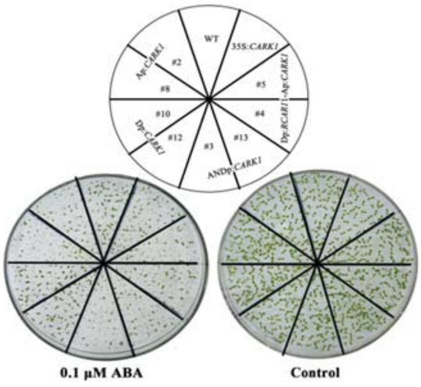

Figure S5. Cotyledon greening assay. Seeds were in MS medium supplemented with or without 0.1 µM ABA, and cotyledon greening rates were recorded after 5 days. The ratio of cotyledon greening was determined for over 70 seeds from three independent experiments.

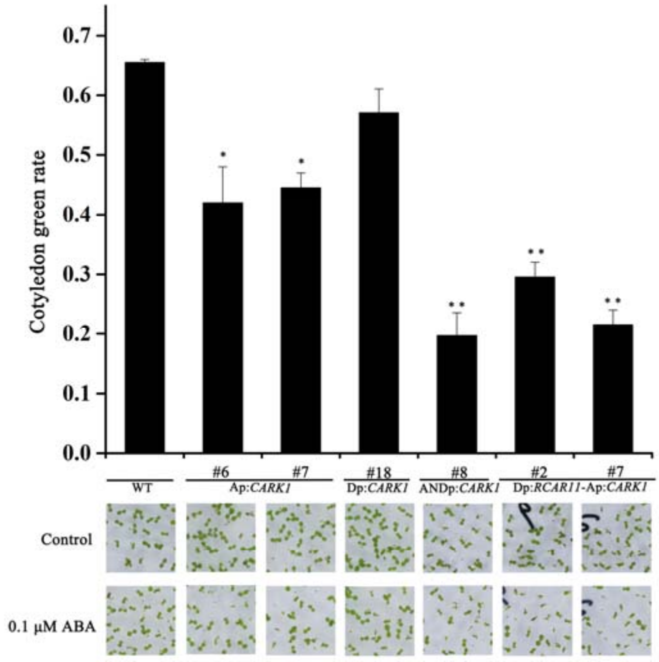

Figure S6. Cotyledon greening assay.

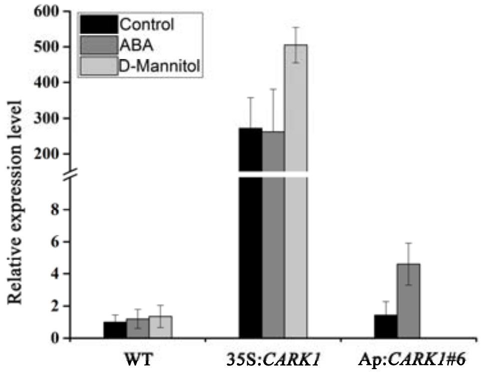

Figure S7. Relative expression levels of total *CARK1* by qRT-PCR analysis. Twelve-day-old seedlings were incubated in MS liquid medium with or without 50 μM ABA for 3 h and 200 mM D-mannitol for 2 h.

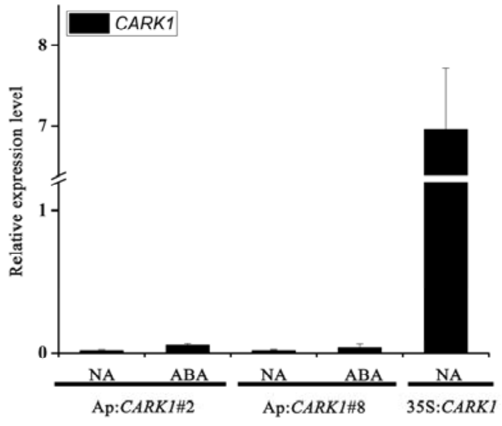

Figure S8. Relative expression levels of exogenous *CARK1* by qRT-PCR analysis. Twelve-day-old seedlings were incubated in MS liquid medium with or without 50 μM ABA for 3 h. The primers were F(CARK1) and R(NOS).

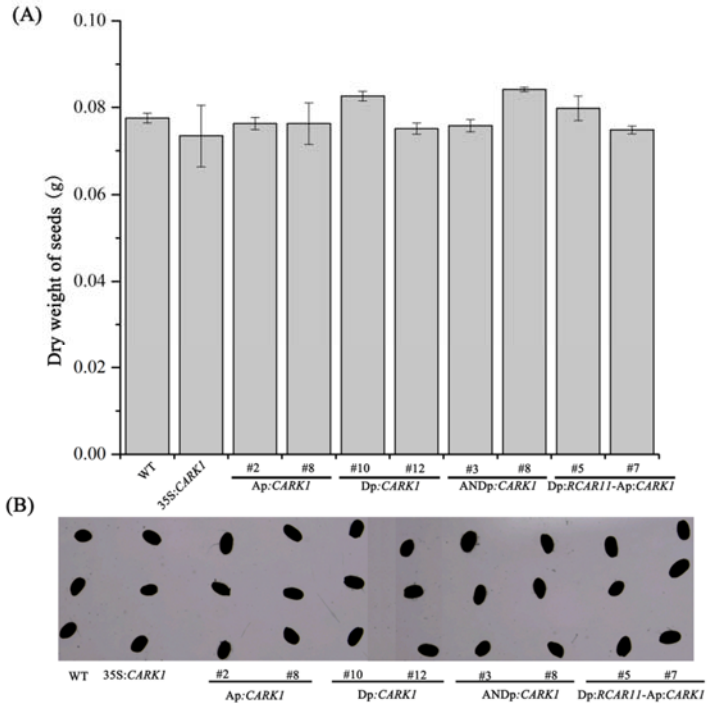

Figure S9. Phenotypic analysis of seeds. **(A) Dry weight of seeds.** Weighing 0.1 mL volume of dried seeds of all transgenic plants under normal growth conditions. **(B) The size of seeds.** The image was taken by the microscope.

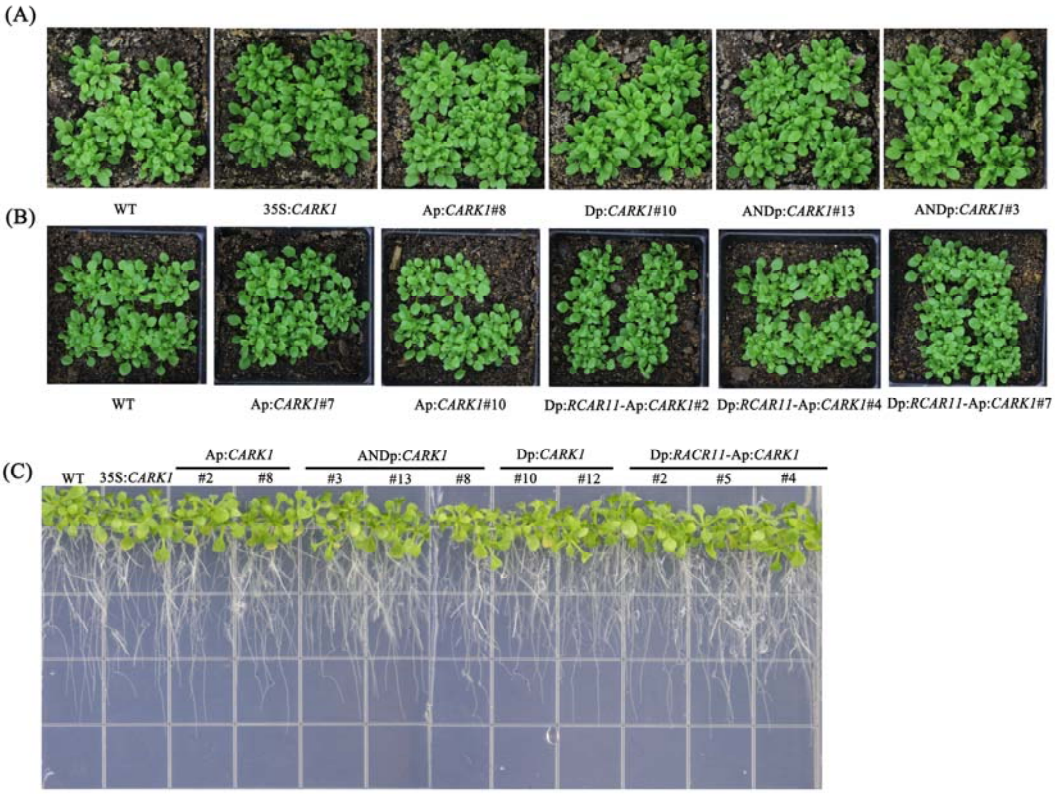

Figure S10. The growth of plants under normal conditions. **(A)** and **(B)** Two-week-old *Arabidopsis thaliana* plants in soil. **(C)** The growth of *Arabidopsis thaliana* plants in MS medium.

Table S1. Survival rate of drought tolerance assay.

	**WT**	**35S:*CARK1***	Ap:*CARK1*					Dp:*CARK1*				ANDp:*CARK1*			Dp:*RCAR11*-Ap:*CARK1*				
			#2	#6	#7	#8	#10	#10	#12	#14	#18	#3	#8	#13	#1	#4	#5	#7	#10
1	0	40%	-	-	-	-	-	0	51%	-	0	100%	100%	100%	-	-	-	-	-
2	39%	36%	29%	0	18%	100%	32%	100%	17%	-	0	67%	100%	100%	-	-	-	-	-
3	0	0	100%	5%	0	100%	0	100%	0	50%	100%	100%	96%	95%	95%	100%	7%	95%	70%
4	0	6%	0	20%	0	0	5%	2%	0	0	30%	85%	100%	100%	100%	100%	90%	100%	96%

Table S2. Primers for assays in this study.

Assays	Vectors/Genes	Primer sequences (5′-3′)
Primers for transgenic plant construction	Dp:*CARK1*	F(*Hind*III):GCCTGTAAGCTTGCACACGA<br>CGTAAACGT
		R(*Xba* I):ATCTAGAGTCCCCCGTGTTCT<br>CTCCAAATG
	Ap:*CARK1*	F(*Hind*III):GCCTGTAAGCTTGCCACGTA<br>GAGAGCAACT
		R(*Xba* I):ATCTAGAGTCCCCCGTGTTCT<br>CTCCAAATG
	ANDp:*CARK1*	F(*Hind*III):GCCTGTAAGCTTGCACACGA<br>CGTAAACGT
		R(*Xba* I):ATCTAGAGTCCCCCGTGTTCT<br>CTCCAAATG
	Dp:*RCA11*-Ap:*CARK1*	F(*Hind*III):GACCATGATTACGCCAAGCT<br>TGCCACACGACGTAAA
		R(*Hind*III):TGCTCTCTACGTGGCAAGCT<br>TGATCTAGTAA CATAG
Primers for qRT-PCR analysis	Total *CARK1*	F:ACCTTCCAGTCATTCAGA
		R:ACCATAGTTATCCGTTATATCC
	Exogenous *CARK1*	F(HA):CCATACGACGTCCCAGACTACG
		R(CARK1):TCGCAATCTCGAAACCATTG<br>ATACCT
	Total *RCAR11*	F:ATCGTCATCAGTGGATTA
		R:TAATTCGTCAGCCTATGT
	Exogenous *RCAR11*	F(RCAR11):ATCGTCATCAGTGGATTA
		R(FLAG):CTTATCGTCGTCATCCTTGTA
	RD29A	F:TCAACACACACCAGCAGCAC
		R:ATCGGAAGACACGACAGGAA
	RD29B	F:ATCGGAAGACACGACAGGAA
		R:TCTCTTTTCGCTTCCCAGT
	ACTIN2/8	F:GGTAACATTGTGCTCAGTGGTGG
		R:AACGACCTTAATCTTCATGCTGC

Table S3. Primers for assays in this study.

Assays	Vectors/Genes	Primer sequences (5′-3′)
Primers for synthetic promoter analysis	Dp:*eGFP*	F(*Hind*III):GCCTGTAAGCTTGCACACGACGTAAACGT
		R(*Xba*I):ATCTAGAGTCCCCCGTGTTCTCTCCAAATG
	Ap:*eGFP*	F(*Hind*III):GCCTGTAAGCTTGCCACGTAGAGAGCAACT
		R(*Xba*I):ATCTAGAGTCCCCCGTGTTCTCTCCAAATG
	ANDp:*eGFP*	F(*Hind*III):GCCTGTAAGCTTGCACACGACGTAAACGT
		R(*Xba*I):ATCTAGAGTCCCCCGTGTTCTCTCCAAATG
	Dp:*FLUC*	F(*Hind*III):GTCGACGGTATCGATAAGCTTGCCACACGACGTAAA
		R(*BamH*I):CGCTCTAGAACTAGTGGATCCGTCCCCCGTGTTCTC
	Ap:*FLUC*	F(*Hind*III):GTCGACGGTATCGATAAGCTTGCCACGTAGAGAGCA
		R(*BamHI*):CGCTCTAGAACTAGTGGATCCGTCCCCCGTGTTCTC
	ANDp:*FLUC*	F(*Hind*III):GTCGACGGTATCGATAAGCTTGCCACACGACGTAAA
		R(*BamH*I):CGCTCTAGAACTAGTGGATCCGTCCCCCGTGTTCTC
	35S:*DREB2A*	F(*Xba*I):GAGAACACGGGGGACTCTAGAATGGCAGTTTATGATCAGAGTGGAGATA
		R(*Sac*I):CGATCGGGGAAATTCGAGCTCTTAGTTCTCCAGATC

Table S4. Dry weight of seeds of all transgenic plants under normal growth conditions.

**Lines**	**Weight (g)**
**WT**	0.0775 ± 0.0011
**35S:*CARK1***	0.0735 ± 0.0070
**Ap:*CARK1* #2**	0.0763 ± 0.0014
**Ap:*CARK1* #8**	0.0763 ± 0.0049
**Dp:*CARK1* #10**	0.0826 ± 0.0011
**Dp:*CARK1* #12**	0.0751 ± 0.0013
**ANDp:*CARK1* #3**	0.0758 ± 0.0014
**ANDp:*CARK1* #8**	0.0841±0.0005
**Dp:*RCAR11*-Ap:*CARK1* #5**	0.0798±0.0028
**Dp:*RCAR11*-Ap:*CARK1* #7**	0.0748 ± 0.0009
